# Safety recommendations on laboratory and blood count values for early mobilization in critically ill adults: an expert consensus

**DOI:** 10.62675/2965-2774.20260058

**Published:** 2026-08-21

**Authors:** Andrés Mauricio Enriquez Popayan, Henry Mauricio Parada-Gereda, Luis Alexander Peña-López, Longxiang Su, Milton Santillán-Zuta, Wilson Mauricio Lozano, Hongbo Luo, Daniel Lago Borges, Darío Salvador Villalba, Romina Amelia Pratto, José M. Cruz-Touzet, Susana Arias-Rivera, Carmen Ana Cerron Cabezas, Rosana Goñi-Viguria, Marta Raurell-Torredá, Debora Stripari Schujmann, Patrick Sepúlveda Barisich, Santiago Nicolás Saavedra, Miguel Ángel Martínez Camacho, Leonardo Alexander Quevedo Florez, Ricardo Arriagada, Alexis Silva-Gutiérrez, Maritza Giovanna Soto Limo, Francisco Jimenez Salazar

**Affiliations:** 1 SES Hospital Universitario de Caldas Hospital Regional de la Orinoquia Departamento de Fisioterapia en Cuidados Intensivos Yopal Colombia Departamento de Fisioterapia en Cuidados Intensivos, SES Hospital Universitario de Caldas - Manizales, Colombia. Grupo de Investigación GIHORO, Hospital Regional de la Orinoquia, Yopal, Colombia.; 2 Unidad de Cuidado Intensivo Clínica Reina Sofía Clinica Colsanitas, grupo de investigación en Nutrición Clinica y Rehabilitación Bogotá Colombia Unidad de Cuidado Intensivo Clínica Reina Sofía, Clinica Colsanitas, grupo de investigación en Nutrición Clinica y Rehabilitación, Keralty - Bogotá, Colombia.; 3 Universidad del Cauca Grupo de Investigación GRIAN Departamento de Anestesiología Popayán Colombia Departamento de Anestesiología, Universidad del Cauca, Popayán, Colombia. Grupo de Investigación GRIAN, Universidad del Cauca. Popayán, Colombia.; 4 Peking Union Medical College Hospital, Peking Union Medical College Chinese of Medical Sciences State Key Laboratory of Complex Severe and Rare Disease Departmet of Critical Care Medicine Beijing China Departmet of Critical Care Medicine, State Key Laboratory of Complex Severe and Rare Disease, Peking Union Medical College Hospital, Peking Union Medical College Chinese of Medical Sciences, Beijing, China.; 5 Universidad Norbert Wiener Facultad de Ciencias de la Salud, Programa de Tecnologia Médica en Terapia Física y Rehabilitación Lima Perú Facultad de Ciencias de la Salud, Programa de Tecnologia Médica en Terapia Física y Rehabilitación, Universidad Norbert Wiener, Lima, Perú.; 6 Universidad Industrial de Santander Escuela de Fisioterapia Bucaramanga Colombia Escuela de Fisioterapia, Universidad Industrial de Santander, Bucaramanga, Colombia.; 7 Unidade de Terapia Intensiva Adulto do Hospital Universitário da Universidade Federal do Maranhão São Luís Brazil Unidade de Terapia Intensiva Adulto do Hospital Universitário da Universidade Federal do Maranhão, São Luís, Brazil; 8 Santa Catalina Neurorehabilitación Clínica Departamento de docencia e investigación Buenos Aires Argentina Departamento de docencia e investigación, Santa Catalina Neurorehabilitación Clínica, Buenos Aires, Argentina.; 9 Servicio de Kinesiología y Rehabilitación, Sanatorio Anchorena Recoleta Buenos Aires Argentina Servicio de Kinesiología y Rehabilitación, Sanatorio Anchorena Recoleta, Buenos Aires, Argentina.; 10 Hospital Guillermo Almenara Departamento de Cuidados Críticos Lima Perú Departamento de Cuidados Críticos. Hospital Guillermo Almenara, Lima, Perú; 11 Hospital Universitario de Getafe CIBER Enfermedades Respiratorias, Instituto de Salud Carlos III Departamento de investigación de enfermería Madrid España Departamento de investigación de enfermería, Hospital Universitario de Getafe, Getafe, España. CIBER Enfermedades Respiratorias, Instituto de Salud Carlos III, Madrid, España.; 12 Hospital Guillermo Almera Departamento de Trasplante Lima Peru Departamento de Trasplante, Hospital Guillermo Almera, Lima, Peru.; 13 Clínica Universidad de Navarra Departamento de Enfermería en Área de Críticos Pamplona España Departamento de Enfermería en Área de Críticos, Clínica Universidad de Navarra, Pamplona, España; 14 Universitat de Barcelona Facultad de Enfermería Departamento de Enfermeria Clinica y Fundamental España Departamento de Enfermeria Clinica y Fundamental, Facultad de Enfermería, Universitat de Barcelona, España.; 15 Universidade de São Paulo Faculdade de Medicina Departamento de Fisioterapia, Ciências da Comunicação e Distúrbios da Comunicação e Terapia Ocupacional São Paulo Brazil Departamento de Fisioterapia, Ciências da Comunicação e Distúrbios da Comunicação e Terapia Ocupacional, Faculdade de Medicina, Universidade de São Paulo - São Paulo, Brazil.; 16 Hospital La Serena Servicio de Kinesiología y Rehabilitación Chile Servicio de Kinesiología y Rehabilitación, Hospital La Serena, Chile.; 17 Clínica Rehabilitación Otamendi Servicio de Kinesiología Respiratoria Buenos Aires Argentina Servicio de Kinesiología Respiratoria, Clínica Rehabilitación Otamendi, Buenos Aires, Argentina.; 18 Hospital General de México "Dr Eduardo Liceaga" Departamento de Fisioterapia en Cuidados Intensivos Ciudad de México México Departamento de Fisioterapia en Cuidados Intensivos, Hospital General de México "Dr Eduardo Liceaga", Ciudad de México, México.; 19 Hospital Universitario Fundación Santa fe de Bogotá Departamento de Medicina Critica y Cuidados Intensivos Bogotá Colombia Departamento de Medicina Critica y Cuidados Intensivos, Hospital Universitario Fundación Santa fe de Bogotá, Bogotá, Colombia.; 20 Unidad de Paciente Critico Adulto Hospital Las Higueras de Talcahuano Chile Unidad de Paciente Critico Adulto, Hospital Las Higueras de Talcahuano, Chile.; 21 Unidad de Cuidados Intensivos Adultos Hospital Clinico Herminda Martin Servicio de Medicina Física y Rehabilitación Chile Servicio de Medicina Física y Rehabilitación, Unidad de Cuidados Intensivos Adultos, Hospital Clinico Herminda Martin, Chile.; 2 Hospital Regional de Lambayaque Departamento de Medicina en Cuidados Intensivos Chiclayo Peru Departamento de Medicina en Cuidados Intensivos, Hospital Regional de Lambayaque, Chiclayo, Peru.; 23 Hospital Univalle, Cochabamba Departamento de Medicina en Cuidados Intensivos Cochabamba Bolivia Departamento de Medicina en Cuidados Intensivos, Hospital Univalle, Cochabamba, Bolivia.

**Keywords:** Early ambulation, Exercise, Critical illness, Laboratory critical values, Patient safety, Respiration, artificial, Clinical decision-making, Consensus

## Abstract

Although early mobilization is a cornerstone of modern critical care for reducing intensive care unit-acquired weakness, the duration of mechanical ventilation, and length of stay, its implementation remains highly variable due to the absence of standardized laboratory criteria to guide clinical decision-making. Therefore, this study aimed to generate safety recommendations regarding laboratory and blood count values to be considered prior to initiating early mobilization in critically ill adults, both with and without invasive supports, across different clinical conditions. To address this evidence gap, a modified Delphi process was conducted with 24 highly experienced intensive care unit staff, including physiotherapists, physicians, and nurses. All panelists met strict criteria related to clinical expertise, advanced training, and scientific productivity. Over eight iterative rounds, the panel achieved at least 85% agreement on 119 items across six clinical domains: respiratory, cardiovascular, renal, surgical, hematological/immunological, and neurological. Key laboratory parameters considered included hemoglobin, platelets, lactate, glucose, international normalized ratio, acid-base state (pH), and potassium in selected clinical contexts. The consensus also provides novel guidance in two previously underexplored areas: first, mobilization during blood product transfusions, recommending postponement of early mobilization until transfusion completion; and second, the importance of dynamic trends in routine laboratory tests, identifying clinically meaningful declines in hemoglobin: ≥ 2g/dL/24 hours and platelets: ≥ 20% within 24 hours as contraindications to mobilization. This consensus offers a pragmatic framework that has the potential to reduce interprofessional variability, enhance patient safety, and inform the development of local and national protocols, including in resource-limited settings. Nonetheless, prospective validation across different healthcare contexts is required to confirm its clinical impact and generalizability.

## INTRODUCTION

Early mobilization (EM) in critically ill patients is a key strategy in the recovery of intensive care unit (ICU) hospitalized individuals.^(1,2)^ Previous studies have demonstrated multiple benefits, including a reduction in the duration of mechanical ventilation and the minimization of ICU-acquired weakness.^(3,4)^

Despite several recommendations supporting its implementation,^(5,6)^ a substantial gap remains in the standardization of clinical criteria. To date, clinical criteria are available across several domains, including cardiovascular, respiratory, neurological, and musculoskeletal systems;^(7)^ however, there are currently no globally accepted guidelines defining laboratory values that determine safety prior to the initiation of EM in ICU patients, resulting in marked variability in clinical judgment among professionals.

Laboratory parameters such as hemoglobin, platelet count, and lactate^(8-10)^ are of critical importance, as they provide essential information regarding physiological stability.^(11-13)^ However, the available literature proposes heterogeneous clinical reference points suggested by different authors. In this context, significant discrepancies have been described in the laboratory thresholds considered safe for mobilization in critically ill patients. For example, acceptable lactate values vary widely across studies, with proposed cut-off points ranging from < 2 millimoles per liter (mmol/L)^(14)^ to < 3mmol/L^(15)^ and even < 6mmol/L.^(16)^ Similarly, recommended hemoglobin levels show variability, with thresholds ranging from >7 grams per decilitre (g/dL)^(17)^ to > 8g/dL.^(18)^ This heterogeneity is also observed in platelet counts, where the suggested minimum values fluctuate between > 20,000 per microlitre (mcL)^(16)^ and > 50,000mcL.^(18)^

These discrepancies generate uncertainty among healthcare professionals and may lead to unnecessary delays or inappropriate initiation of mobilization, potentially compromising patient safety or increasing variability in clinical decision-making. Moreover, given that critical illness requires an individualized and condition-specific approach, and that each pathology exhibits distinct clinical behavior, EM should be tailored from the initial assessment phase, incorporating the evaluation of laboratory parameters into clinical decision-making.^(19)^

This evidence gap underscores the need to develop consensus-based recommendations to guide clinical practice and reduce heterogeneity in decision-making. Therefore, this study aimed to generate safety recommendations regarding laboratory and blood count values to be considered prior to initiating EM in critically ill adults, both with and without invasive supports, across different clinical conditions.

## METHODS

### Study design

A consensus study was conducted using a modified Delphi methodology.^(20,21)^ The use of a modified Delphi approach is well aligned with the present study, given the limited and heterogeneous nature of the available evidence on laboratory safety thresholds for EM in critically ill patients. Existing studies report inconsistent reference values, often fragmented across clinical contexts, hindering the development of standardized recommendations. In this context, the modified Delphi method enables the systematic integration of expert judgment through iterative rounds and controlled feedback, making it particularly well-suited to inform clinical decision-making in areas with limited evidence.

The panel comprised a multidisciplinary group (physiotherapists, physicians, nurses) of experts selected according to predefined criteria that ensured both representativeness and expertise in critical care. These criteria were defined and benchmarked against recommendations available in the literature.

### Expert selection criteria

Expert selection criteria included: more than 10 years of experience in critical care;^(22)^ advanced postgraduate training (specialty, Master's degree, or Doctorate) in related fields;^(23)^ authorship of at least one scientific research article;^(24)^ participation as a speaker or lecturer in at least two academic events within the past 5 years.^(25)^

These criteria ensured the inclusion of professionals with a solid career trajectory, a comprehensive perspective, and the scientific competence required to formulate recommendations.

### Recruitment strategy

Potential experts were identified through targeted searches of academic networks and scientific societies related to critical care. Eligible experts were contacted individually via email.

### Consensus development

#### Phase 1: Literature review

Two independent investigators conducted a structured literature search in PubMed®, ScienceDirect, *Literatura Latino-Americana e do Caribe em Ciências da Saúde* (Lilacs), the Cochrane Library, Europe PMC, and Scopus, from database inception to June 2025. Additionally, a manual search of the reference lists of included studies was performed to identify potentially relevant articles.

Although this process followed systematic search principles, it was not intended as a formal systematic review but rather as an evidence-informed scoping approach to identify relevant laboratory parameters for the Delphi process.

The screening process was conducted using Rayyan,^(26)^ allowing independent and blinded study selection. Titles and abstracts were initially screened, followed by full-text assessment of potentially eligible studies. Discrepancies were resolved by consensus or, when necessary, through consultation with a third reviewer.

Eligible studies included randomized controlled trials, prospective and retrospective cohort studies, and systematic reviews that evaluated laboratory parameters in relation to the safety or feasibility of EM in critically ill patients. Studies that did not address laboratory parameters or were not related to EM in the ICU setting were excluded.

Given that the available evidence on specific laboratory thresholds is limited and heterogeneous, the review was primarily oriented towards identifying the most frequently reported laboratory parameters and the range of values suggested across different clinical contexts, rather than establishing standardized cut-off points.

This review allowed the identification of laboratory tests most frequently reported in relation to EM safety, the compilation of suggested value ranges, and the definition of the initial parameters included as response options in the Delphi questionnaire.

The search strategy combined controlled vocabulary and free-text terms related to EM, laboratory parameters, and critical care. Example search terms included:

"early mobilisation" OR "early mobilization" OR "mobilisation" OR "mobilization".

"Intensive care unit" OR "ICU" OR "critical care".

"Laboratory parameters" OR "laboratory values" OR "biomarkers".

"hemoglobin" OR "haemoglobin" OR "platelet count "OR "platelets" OR "lactate".

"safety" OR "clinical thresholds" OR "cut-off values" OR "reference values".

The final search strategy was adapted for each database by combining these terms with Boolean operators (AND/OR) according to each database's specific indexing system.

#### Phase 2: Delphi questionnaire design

Based on the preliminary review, two investigators developed the questionnaire using Google Forms. The form included information on confidentiality and the study's purpose, instructions for rating each laboratory parameter, and multiple-choice questions to quantitatively assess the level of agreement.

For each parameter (hemoglobin, platelets, lactate, glucose, international normalized ratio, acid-base state (pH), and potassium), three to four potential reference levels were presented as safety criteria for active EM, active-assisted EM, or contraindication. These categories were predefined and remained visible throughout the questionnaire.

Experts responded to six different clinical scenarios: respiratory, cardiovascular, renal, surgical, hematological/immunological, and neurological, each accompanied by clinical reference points. The response options were generated based on the different cut-off points identified in the literature and were complemented by options proposed at the investigators’ discretion. Each section included an open-ended field for comments or suggestions. The observations suggested by the experts were shared anonymously as comments in the subsequent rounds.

The initial questionnaire consisted of 111 items distributed across all defined sections and parameters. The document was structured into individual sections, each dedicated to a specific predominant pathology. This was followed by a section focusing on the transfusion of blood products during EM. Finally, the document concluded with a section addressing the follow-up of routine laboratory tests.

#### Phase 3: Delphi process

Professionals who met the selection criteria and voluntarily agreed to participate received detailed information about the study objectives and the functioning of the Delphi method. To ensure anonymity and reduce the risk of bias, each participant responded independently under an assigned numerical code and had no access to other experts’ responses or identities.

Consensus was defined as ≥ 85% agreement among participants for a given item. This threshold was established based on prior literature, where agreement levels within this range are widely recognized as indicative of a high level of consensus.^(27)^

Voting rounds were conducted electronically. Items that achieved consensus were considered definitive and excluded from subsequent rounds. Items that did not reach consensus were redistributed in successive rounds, accompanied by a statistical summary (median, interquartile range, and percentage of agreement), allowing experts to reconsider their individual judgments.

Operational coordination was undertaken by a member of the research team who was not part of the expert panel to preserve the independence of the process. This individual managed questionnaire distribution and follow-up and performed initial data cleaning.

Data were exported to a Microsoft Excel database for storage and analysis. The entire Delphi process lasted three months, from the first to the final round, with predefined deadlines for response submission and review.

Qualitative comments provided by panel members during each Delphi round were analyzed using an iterative thematic approach. The research team reviewed and synthesized the feedback to identify recurring themes and areas of disagreement, which informed item refinement, clarification of ambiguities, and the design of subsequent Delphi rounds, while preserving the intent of the expert input.

### Interpretation of laboratory tests consensus

The laboratory thresholds presented are expressed using boundary values (≥ or ≤) rather than continuous intervals. When a threshold includes the symbol ≥, it indicates that values equal to or above the specified cut-off are considered safe for initiating EM in relation to that laboratory parameter. Conversely, when the symbol ≤ is used, values equal to or below the specified limit indicate that EM should not be initiated. When both limits are present, the interval between them represents the range considered safe for initiating EM. This approach was applied consistently across all laboratory parameters presented.

Values falling between defined thresholds should be interpreted with caution and in conjunction with clinical judgment, as they may represent transitional or context-dependent states rather than strict categorical cut-offs.

### Ethical considerations

This study complied with international guidelines, including the Declaration of Helsinki, the Nuremberg Code, and Colombian research regulations regarding informed consent, data protection, and risk classification (Resolution 8430 of 1993, Ministry of Health). The study was approved by the Health Research Ethics Committee (CEIS) of the Hospital Regional de la Orinoquía (Minutes No. 011, 20 June 2025).

### Statistical analysis

Data analysis was performed using descriptive statistics, in accordance with the nature of the Delphi method. All responses were exported from Google Forms and organized in a Microsoft Excel database, then processed and analyzed using R software version 4.4.2.

For each questionnaire item, the following measures were calculated: percentage of agreement for each response option; median and interquartile range (IQR) of ratings, where applicable (Likert scale 1 - 9); absolute and relative frequencies for selected clinical reference point categories; and identification of consensus, defined as ≥ 85% agreement among experts.

In each round, items that met the consensus criterion were excluded from analysis in subsequent rounds. Items without consensus were descriptively reanalyzed, and their results were summarized to provide participants with feedback in the following round, using measures of central tendency and dispersion.

Qualitative comments provided in open-ended fields were analyzed using simple thematic categorization to identify convergent patterns relevant to the final interpretation of the results.

## RESULTS

A total of 24 experts were recruited, and all completed every round of the Delphi process. The sample showed a predominance of male participants (66.7%), while female participants represented 33.3%. Experts were drawn from ten countries, with Colombia and Peru each accounting for 16.7% of participants. Argentina, Chile, and Spain contributed 12.5% each, while Brazil and China accounted for 8.3%, and Mexico, Bolivia, and the Netherlands each represented 4.2%.

The sample was composed primarily of physiotherapists (58.3%), followed by physicians (25.0%) and nurses (16.7%) (Table 1). All participants had advanced training in the field: 75% were ICU specialists, 8% held a cardiopulmonary specialty, 8% had a Master's degree, and 8% held a Doctoral degree. Additionally, 46% of experts had at least two advanced qualifications, and 17% held all three levels. Median professional experience was 18 years (IQR 15 - 26), with a median of 16 years of ICU experience (IQR 13 - 20). The median number of publications was 14 (IQR 7 - 40), and the median number of academic events as speakers in the past five years was 16 (IQR 6 - 21) (Table 1).

**Table 1:** Characteristics of the participating experts

#	Gender	Country	Profession	Areas of professional expertise	Postgraduate training	Years employment	ICU years	# Articles	# Events
1	M	Colombia	Pt	Clinical, teaching, research	Specialty in ICU, Master's	20	19	12	6
2	M	Colombia	Pt	Clinical, teaching, research, management	Specialty in ICU, Master's	32	16	8	30
3	M	Colombia	Pt	Clinical, teaching, research	Specialty in ICU, Master's, Doctorate	16	15	14	10
4	M	Argentina	Pt	Clinical, teaching, research	Specialty in ICU	22	22	40	15
5	F	Argentina	Pt	Clinical, teaching, research, management	Specialty in ICU	17	15	15	5
6	M	Argentina	Pt	Clinical, teaching, research	Specialty CR	14	14	14	3
7	M	Chile	Pt	Clinical, teaching, research	Specialty in ICU, Master's	13	13	21	20
8	M	Chile	Pt	Clinical, research	Specialty in ICU, Master's, Doctorate	15	15	6	8
9	M	Chile	Pt	Clinical, teaching, research, management	Specialty in ICU, Master's	14	13	7	25
10	M	Brazil	Pt	Clinical, teaching, research	Specialty in ICU, Master's, Doctorate	21	20	50	25
11	F	Brazil	Pt	Clinical, teaching, research	Specialty in ICU, Doctorate	16	16	13	20
12	M	Perú	Pt	Clinical, teaching, research	Specialty in CR, Master's	25	20	5	20
13	M	México	Pt	Clinical, teaching, research, management	Specialty in ICU, Master's, Doctorate	13	11	33	22
14	M	Netherlands	Pt	Clinical, teaching, research, management	Doctorate in ICU	45	45	285	50
15	M	Perú	Ph	Clinical, teaching	Specialty in ICU	32	25	2	10
16	F	Perú	Ph	Clinical	Specialty in ICU	12	11	10	3
17	F	Perú	Ph	Clinical, teaching	Specialty in ICU, Master's	20	13	1	3
18	M	Colombia	Ph	Clinical, teaching, research, management	Specialty in ICU, Master's	16	10	11	7
19	M	Bolivia	Ph	Clinical, teaching	Specialty in ICU, Master's	15	10	1	3
20	M	China	Ph	Clinical, teaching, research, management	Doctorate in ICU	20	15	200	100
21	F	Spain	Nur	Clinical, research, and management	Master's in ICU	36	27	60	5
22	F	Spain	Nur	Clinical, teaching, research	Master's in ICU, Doctorate	31	16	65	18
23	F	Spain	Nur	Clinical	Specialty in ICU, Master's	28	28	40	16
24	F	China	Nur	Clinical, teaching	Specialty in ICU	19	19	4	21

# - number; ICU - intensive care unit; M - male; Pt - physiotherapists; F - female; CR - cardiorespiratory; Ph- physicians; Nur - nurses.

The initial questionnaire items were derived from the literature search described in the Methods section and served as the basis for the first Delphi round.

### Consensus development: Delphi process

**Figure 1:**
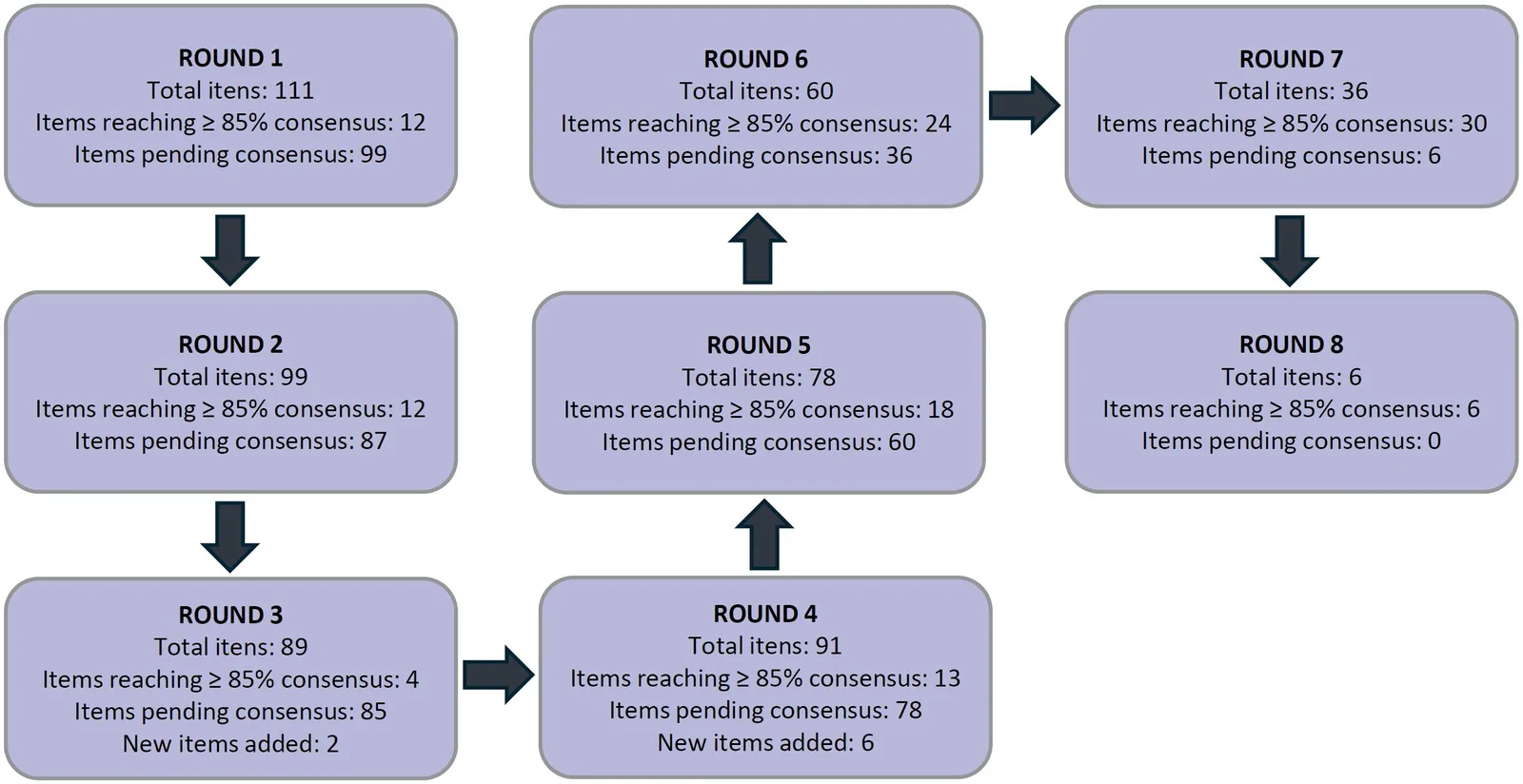
Flow diagram of the Delphi consensus rounds.

A total of eight rounds were conducted until ≥ 85% agreement was achieved across all items. New items proposed during rounds 3 and 4 were incorporated, resulting in a final questionnaire of 119 items. Only items reaching the predefined consensus threshold were retained (Figure 1). A 100% response rate was maintained across all rounds. The final percentage of agreement achieved for each item evaluated throughout the Delphi process is available in the Supplementary Material.

A color-coded system was established to facilitate interpretation of EM safety levels: active EM (green), active-assisted EM (yellow), and contraindication (red) (Table 2).

**Table 2:** Traffic-light coding of the main operational concepts

EM active	This refers to a situation in which the patient has an adequate level of consciousness, understands commands, and can execute them autonomously. In this context, the patient performs physical activities independently
EM active-assisted	A process in which the patient requires assistance due to their level of consciousness, clinical condition, or neuromuscular limitations. In this context, the patient attempts to perform movements independently but requires the support of one or more healthcare professionals or instrumental devices to carry out the activities safely and effectively
Contraindication	It is not considered safe and constitutes a contraindication to initiating active or active-assisted EM in critically ill adult patients, both with and without invasive supports

EM - early mobilization.

Safety recommendations were organized into six clinical domains: respiratory, cardiovascular, renal, surgical, hematological/immunological, and neurological. Across these domains, consensus was reached on key laboratory parameters, including hemoglobin, platelets, lactate, glucose, international normalized ratio, and pH. Potassium was included only in selected clinical contexts.

**Figure 2:**
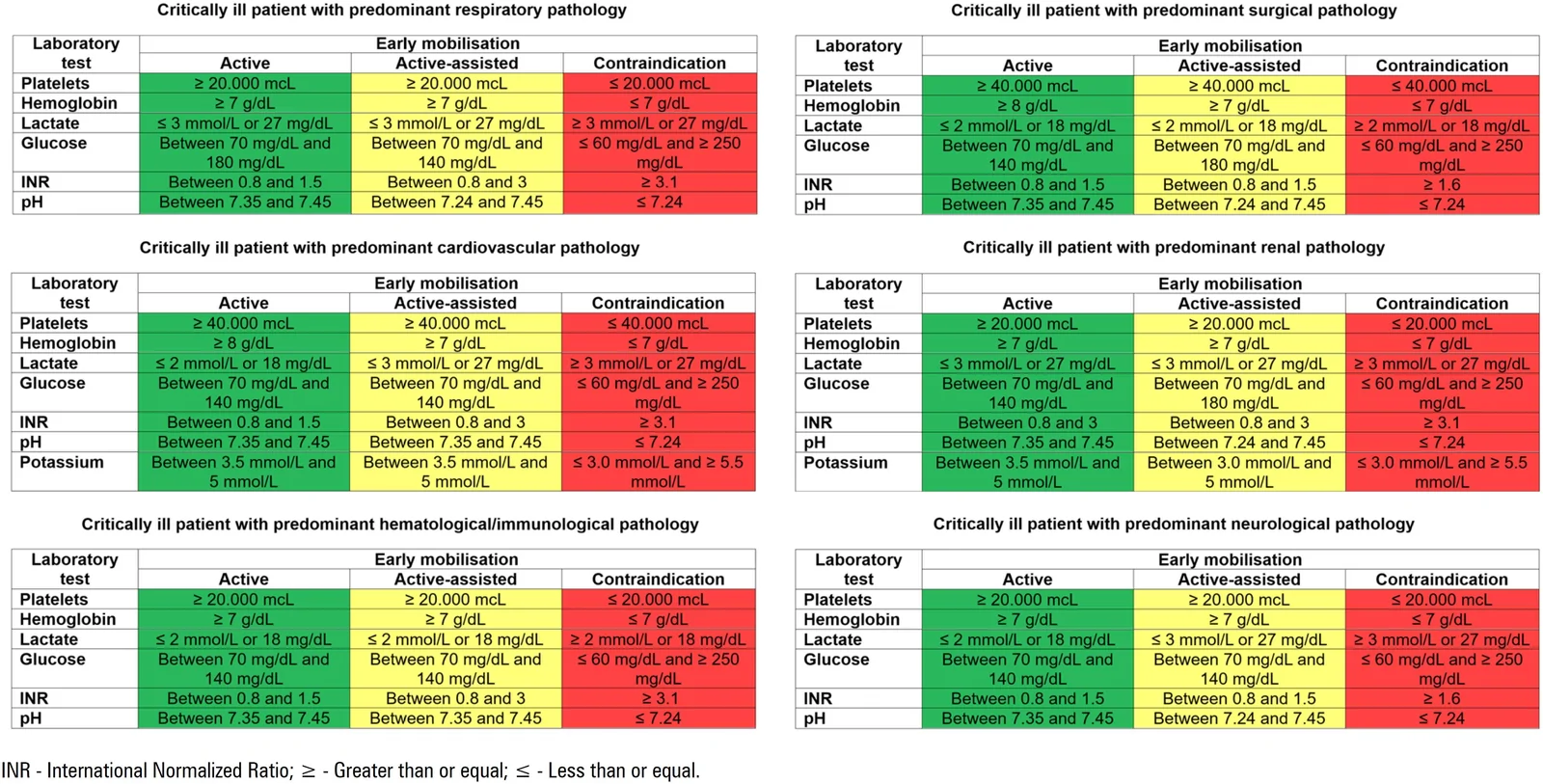
Safety recommendations on laboratory and blood count values for early mobilization in critically ill adults, both with and without invasive supports.

Source: Author's own Delphi consensus.

Common platelet ranges were identified across several domains during active and active-assisted EM. Hemoglobin thresholds were consistent across all domains for contraindication, as observed for acid-base status (pH) during active EM (Figure 2).

Variation in laboratory reference values was observed in parameters such as lactate, international normalized ratio, and platelet count, reflecting differences in clinical decision-making contexts. For example, a higher lactate threshold may be considered acceptable in a hemodynamically stable postoperative patient. In contrast, a more conservative threshold may be preferred in a patient with ongoing shock, reflecting differences in physiological reserve and clinical context.

Recommendations for EM during blood product transfusion are summarized in table 3, while guidance on routine laboratory monitoring is presented in table 4.

**Table 3:** Early mobilization in the context of transfusions

Transfusion context	Recommendation
Do you consider it safe to initiate active or active-assisted EM in a critically ill adult patient, both with and without invasive supports, during the administration of a red blood cell transfusion?	No. It is recommended to wait until the transfusion is complete before initiating EM
Do you consider it safe to initiate active or active-assisted EM in a critically ill adult patient, both with and without invasive supports, during the administration of a platelet transfusion?	No. It is recommended to wait until the transfusion is complete before initiating EM
Do you consider it safe to initiate active or active-assisted EM in a critically ill adult patient, both with and without invasive supports, during the administration of a plasma transfusion?	No. It is recommended to wait until the transfusion is complete before initiating EM

EM - early mobilization.

**Table 4:** Follow-up to routine laboratory tests

Routine examination	Recommendation
About routine laboratory tests, how much should hemoglobin decrease over 24 hours to contraindicate the initiation of active or active-assisted EM in critically ill patients, both with and without invasive supports?	A decrease of ≥ 2g/dL compared with the previous day's value
About routine laboratory tests, how much should the platelet count decrease over 24 hours to contraindicate the initiation of active or active-assisted EM in critically ill patients, both with and without invasive supports?	A decrease of ≥ 20% compared with the previous day's value

EM - early mobilization; ≥ - Greater than or equal; g – grams; dL – deciliter; % - percentage.

## DISCUSSION

Although there are currently documents that offer safety recommendations, they do not provide detailed laboratory-based thresholds or criteria to support their systematic implementation in routine clinical practice. To our knowledge, this international consensus is among the first efforts to define laboratory-informed criteria for initiating EM in critically ill adults. High levels of agreement were achieved across all items within the six clinical domains, as well as in transfusion and follow-up scenarios, thereby helping to address this evidence gap and inform clinical decision-making in intensive care practice.

One of the main contributions of this consensus is the incorporation of context-specific and individualized criteria. Jackson et al.^(28)^ highlight that the care of critically ill patients requires a meticulous approach, from initial assessment through to dosing. Accordingly, our study categorizes patients into six diagnostic groups rather than proposing a universal set of laboratory thresholds, allowing each group to reflect a predominant clinical condition and supporting more context-informed decision-making related to EM.

Although the available evidence in this field remains limited, our findings are broadly consistent with previous studies. Regarding platelet count, Sepúlveda et al.^(29)^ recommend a minimum threshold of 20,000 platelets per microliter for initiating EM, a value supported across several clinical domains in our consensus. Similarly, Yang et al.^(30)^ identify hemoglobin levels below 7g/dL as a contraindication, which also showed consistent agreement. However, discrepancies were observed for other biomarkers, such as lactate. Presneill et al.^(31)^ permitted EM at levels below 4mmol/L, whereas our consensus supported a more conservative range between 2 and 3mmol/L, which may reflect differences in clinical context and institutional practices.

Another relevant contribution concerns recommendations for EM during blood product transfusions. Raasveld et al.^(32)^ report that red blood cell transfusion is common among ICU patients, with rates ranging from 25 - 80% depending on the setting. Despite this, current literature and guidelines provide limited guidance on mobilization safety during transfusion. Existing recommendations emphasize close monitoring before, during, and after transfusion, particularly during the initial minutes, to detect adverse reactions and ensure patient safety.^(33-35)^ In this context, our consensus provides structured, operational guidance for a common clinical scenario. Additionally, incorporating dynamic changes in laboratory values – such as trends in hemoglobin and platelet counts – is an important contribution, as it supports a more anticipatory, trajectory-based approach to clinical decision-making.

These recommendations also provide operational clarity for this frequent clinical scenario. Likewise, the recommendations concerning variations in routine laboratory tests associated with EM represent an additional contribution to the current evidence base. These recommendations consider not only absolute values but also trends that may anticipate physiological deterioration before parameters exceed critical ranges, thereby supporting a more dynamic and clinically informed approach to decision-making that integrates patient trajectories.

Regarding specific recommendations in this field, few publications are available. Consensus statements developed by Hodgson et al.,^(36)^ Raurell-Torredà et al.,^(37)^ and Schaller et al.^(38)^ mainly address global safety recommendations, specific subpopulations, and patient positioning, respectively. In contrast, our manuscript focuses on laboratory-informed aspects relevant to daily clinical practice and aims to complement previous consensus statements in a coherent and additive manner.

From a methodological perspective, McMillan et al.^(39)^ report that consensus methods across different disciplines have proven useful when available evidence is variable, fragmented, or limited. In this context, the variability and scarcity of studies on laboratory thresholds – often with divergent recommendations, as reported for hemoglobin^(40,41)^ and platelets^(42,43)^ – support the use of the Delphi method as an approach to synthesize expert judgment and provide structured guidance in areas of uncertainty. Similarly, although reviews focusing on safety recommendations for initiating EM exist, such as those by Nydahl et al.^(44)^ and Adler et al.,^(45)^ these do not detail or include criteria related to laboratory tests. Only a few reviews suggest specific reference values for certain tests;^(28,46)^ however, none comprehensively integrate all the laboratory parameters considered in our consensus. Consequently, the Delphi method was considered appropriate, as it achieved a high level of agreement (≥ 85%) across the items analyzed. This outcome reflects the consistency of expert agreement within this panel. It suggests a perceived need within the clinical community to standardize criteria that may reduce variability in practice, given the inconsistencies and gaps in the available literature on laboratory-based safety criteria for mobilization. These findings support the role of structured expert consensus as a methodological approach to inform clinical decision-making in contexts of uncertainty.

Furthermore, the development of a consensus by an international panel of experts represents a relevant contribution. Byrne et al.^(47)^ note that the participation of experts from different countries allows a consensus to reflect broader perspectives and reduces biases specific to a single national context. In our case, representation from ten countries may help to mitigate biases related to local practice patterns or cultural approaches. Kansal et al.^(48)^ state that multidisciplinary consensus implies strong professional endorsement. In our study, the panel's multidisciplinary composition may reflect a range of perspectives, as supported by the rigorous selection criteria applied. The high level of intensive care experience, continuous training, scientific output, and participation in academic activities are recognized characteristics of expertise, as expert judgment integrates clinical experience, specialized knowledge, and research, thereby supporting the development of these recommendations.

The clinical impact of our research lies in providing structured, clear, and easy-to-interpret information applicable across diverse hospital settings, including those with limited infrastructure or technological resources. The recommendations from this consensus may be useful for both students and clinicians who routinely apply EM. Their implementation may support safer patient selection and more consistent clinical decision-making, potentially reducing variability in practice and improving the structure of mobilization strategies. In addition, these recommendations may facilitate the development of institutional protocols that reduce interprofessional variability. This work can therefore support clinical decision-making and represents a step towards more consistent and patient-centered practice in critical care. It is important to emphasize that standardization of laboratory criteria does not replace comprehensive clinical judgment but rather complements it by providing an objective framework to support decision-making in uncertain scenarios. Finally, this consensus highlights areas for future research, particularly regarding the prospective validation of the proposed thresholds, including mobilization-associated adverse events.

Although this consensus represents a relevant contribution due to its international scope and the high level of expertise of its panel, several limitations should be acknowledged. First, there are currently no controlled trials that precisely define which specific value of each laboratory test corresponds to a completely safe threshold for clinical decision-making. The available evidence is derived mainly from observational studies, reviews, or case reports. Therefore, future research should prospectively validate these proposed thresholds and explore their applicability across larger, more diverse populations. Second, laboratory reference values may vary across institutions due to differences in analytical methods, equipment, and locally defined ranges, which may affect the applicability of the proposed thresholds. Nevertheless, this consensus should be interpreted in light of these limitations, as its primary purpose is to provide a structured clinical framework in the absence of definitive evidence. In addition, the selection of experts may have introduced selection bias, and although the panel included participants from multiple countries, its geographical representation may not fully reflect global practice patterns.

## CONCLUSION

The recommendations derived from this consensus, based on laboratory-informed thresholds from various laboratory and blood count parameters as safety criteria for early mobilization in critically ill adult patients, may support clinical decision-making and help to optimize rehabilitation processes in the intensive care unit.

Nevertheless, further research is required to validate the safety and applicability of the proposed thresholds through studies using diverse methodologies, larger samples, and populations with different pathological conditions.

## Data Availability

The contents underlying the research text are included in the manuscript.
