# Supplementary material for: Safety recommendations on laboratory and blood count values for early mobilization in critically ill adults: an expert consensus
Source: Crit Care Sci. 2026 Aug 21;38:e20260058. doi: 10.62675/2965-2774.20260058 (PMC13518028; doi:10.62675/2965-2774.20260058)
Supplement: Supplementary file 1 [file 2965-2774-ccsci-38-e20260058-Supp01.pdf]

Critical Care Science
https://doi.org/10.62675/2965-2774.20260058

SUPPLEMENTARY MATERIAL

Safety recommendations on laboratory and blood count values for early mobilization in critically ill adults: an expert consensus

Andrés Mauricio Enriquez Popayan[1], Henry Mauricio Parada-Gereda[2], Luis Alexander Peña-López[3], Longxiang Su[4], Milton Santillán-Zuta[5], Wilson Mauricio Lozano[6], Hongbo Luo[4], Daniel Lago Borges[7], Darío Salvador Villalba[8], Romina Amelia Pratto[9], José M. Cruz-Touzet[10], Susana Arias-Rivera[11], Carmen Ana Cerron Cabezas[12], Rosana Goñi-Viguria[13], Marta Raurell-Torredá[14], Debora Stripari Schujmann[15], Patrick Sepúlveda Barisich[16], Santiago Nicolás Saavedra[17], Miguel Ángel Martínez Camacho[18], Leonardo Alexander Quevedo Florez[19], Ricardo Arriagada[20], Alexis G. Silva[21], Maritza Giovanna Soto Limo[22], Francisco Jimenez Salazar[23]

DELPHI PROCESS: FINAL PERCENTAGE OF AGREEMENT ACHIEVED FOR EACH ITEM EVALUATED

FIRST ROUND - Consensus items: 12

Critically ill patient with predominantly respiratory pathology
• Contraindication to initiating active and active-assisted early mobilization
Glucose: ≤ 60mg/dL and ≥ 250mg/dL. Agreement: 100%
pH: ≤ 7.24. Agreement: 96.6%

Critically ill patient with predominantly cardiovascular pathology
• Contraindication to initiating active and active-assisted early mobilization
Glucose: ≤ 60mg/dL and ≥ 250mg/dL. Agreement: 100%
pH: ≤ 7.24. Agreement: 100%

Critically ill patient with predominantly renal pathology
• Contraindication to initiating active and active-assisted early mobilization
Glucose: ≤ 60mg/dL and ≥ 250mg/dL. Agreement: 100%
pH: ≤ 7.24. Agreement: 96.6%

Critically ill patient with predominantly surgical pathology
• Contraindication to initiating active and active-assisted early mobilization
Glucose: ≤ 60mg/dL and ≥ 250mg/dL. Agreement: 100%
pH: ≤ 7.24. Agreement: 100%

Critically ill patient with predominantly hematological/immunological pathology
• Contraindication to initiating active and active-assisted early mobilization
Glucose: ≤ 60mg/dL and ≥ 250mg/dL. Agreement: 100%
pH: ≤ 7.24. Agreement: 96.6%

Critically ill patient with predominantly neurological pathology
• Contraindication to initiating active and active-assisted early mobilization
Glucose: ≤ 60mg/dL and ≥ 250mg/dL. Agreement: 100%
pH: ≤ 7.24. Agreement: 96.6%

This is an open access article under the CC BY license (https://creativecommons.org/licenses/by/4.0/).

SECOND ROUND - Consensus items: 12

Critically ill patient with predominantly respiratory pathology
• Active early mobilization
Hemoglobin: ≥ 7g/dL. Agreement: 96.6%

Critically ill patient with predominantly cardiovascular pathology
• Active early mobilization
pH: Between 7.35 and 7.45. Agreement: 100%

Critically ill patient with predominantly renal pathology
• Active early mobilization
Glucose: Between 70mg/dL and 140mg/dL. Agreement: 87.3%
pH: Between 7.35 and 7.45. Agreement: 96.6%

Critically ill patient with predominantly surgical pathology
• Active early mobilization
pH: Between 7.35 and 7.45. Agreement: 87.3%

Critically ill patient with predominantly hematological/immunological pathology
• Active early mobilization
Glucose: Between 70mg/dL and 140mg/dL. Agreement: 87.3%
pH: Between 7.35 and 7.45. Agreement: 91.5%
• Active-assisted early mobilization
Glucose: Between 70mg/dL and 140mg/dL. Agreement: 91.5%

Critically ill patient with predominantly neurological pathology
• Active early mobilization
Lactate: ≤ 2mmol/L or 18mg/dL. Agreement: 91.5%
Glucose: Between 70mg/dL and 140mg/dL. Agreement: 96.6%
pH: Between 7.35 and 7.45. Agreement: 91.5%
• Active-assisted early mobilization
Glucose: Between 70mg/dL and 140mg/dL. Agreement: 100%

THIRD ROUND - Consensus items: 4

Critically ill patient with predominantly respiratory pathology
• Active-assisted early mobilization
Hemoglobin: ≥ 7g/dL. Agreement: 87.3%

Critically ill patient with predominantly renal pathology
• Active early mobilization
Hemoglobin: ≥ 7g/dL. Agreement: 87.3%
• Active-assisted early mobilization
Hemoglobin: ≥ 7g/dL. Agreement: 100%

Critically ill patient with predominantly surgical pathology
• Active early mobilization
Hemoglobin: ≥ 8g/dL. Agreement: 96.6%

FOURTH ROUND - Consensus items: 13

Critically ill patient with predominantly respiratory pathology
• Active early mobilization
Glucose: Between 70mg/dL and 140mg/dL. Agreement: 87.3%
• Active-assisted early mobilization
Glucose: Between 70mg/dL and 140mg/dL. Agreement: 100%

Critically ill patient with predominantly cardiovascular pathology
• Active early mobilization
Glucose: Between 70mg/dL and 140mg/dL. Agreement: 100%
• Active-assisted early mobilization
Glucose: Between 70mg/dL and 140mg/dL. Agreement: 91.5%
Critically ill patient with predominantly renal pathology
• Active-assisted early mobilization
Glucose: Between 70mg/dL and 180mg/dL. Agreement: 91.5%
• Contraindication to initiating active and active-assisted early mobilization
Potassium: ≤ 3.0mmol/L and ≥ 5.5mmol/L. Agreement: 87.3%

Critically ill patient with predominantly surgical pathology
• Active early mobilization
Glucose: Between 70mg/dL and 140mg/dL. Agreement: 91.5%
• Active-assisted early mobilization
Hemoglobin: ≥ 7g/dL. Agreement: 87.3%
Glucose: Between 70mg/dL and 140mg/dL. Agreement: 87.3%

Critically ill patient with predominantly hematological/immunological pathology
• Active early mobilization
Hemoglobin: ≥ 7g/dL. Agreement: 87.3%
• Active-assisted early mobilization
Hemoglobin: ≥ 7g/dL. Agreement: 87.3%

Critically ill patient with predominantly neurological pathology
• Active early mobilization
Hemoglobin: ≥ 7g/dL. Agreement: 87.3%
• Active-assisted early mobilization
Hemoglobin: ≥ 7g/dL. Agreement: 87.3%

FIFTH ROUND - Consensus items: 18

Critically ill patient with predominantly respiratory pathology
• Active early mobilization
INR: Between 0.8 and 1.5. Agreement: 87.3%
• Contraindication to initiating active and active-assisted early mobilization
Hemoglobin: ≤ 7g/dL. Agreement: 87.3%

Critically ill patient with predominantly cardiovascular pathology
• Contraindication to initiating active and active-assisted early mobilization
Hemoglobin: ≤ 7g/dL. Agreement: 87.3%

Potassium: ≤ 3.0mmol/L and ≥ 5.5mmol/L. Agreement: 91.5%
INR: ≥ 3.1. Agreement: 87.3%

Critically ill patient with predominantly renal pathology
• Active early mobilization
INR: Between 0.8 and 3.0. Agreement: 87.3%
• Active-assisted early mobilization
Potassium: Between 3.0mmol/L and 5.0mmol/L. Agreement: 91.5%
• Contraindication to initiating active and active-assisted early mobilization
Hemoglobin: ≤ 7g/dL. Agreement: 87.3%

Critically ill patient with predominantly surgical pathology
• Active early mobilization
INR: Between 0.8 and 1.5. Agreement: 100%
• Contraindication to initiating active and active-assisted early mobilization
Hemoglobin: ≤ 7g/dL. Agreement: 91.5%

Critically ill patient with predominantly hematological/immunological pathology
• Active early mobilization
INR: Between 0.8 and 1.5. Agreement: 100%
• Contraindication to initiating active and active-assisted early mobilization
Hemoglobin: ≤ 7g/dL. Agreement: 87.3%

Critically ill patient with predominantly neurological pathology
• Active early mobilization
INR: Between 0.8 and 1.5. Agreement: 91.5%
• Active-assisted early mobilization
INR: Between 0.8 and 1.5. Agreement: 87.3%

Early mobilization in the context of transfusions
Do you consider it safe to initiate active or active-assisted early mobilization in a critically ill adult, with or without invasive support, during the administration of a platelet transfusion?
Recommendation: No: it is recommended to wait until the transfusion has finished before initiating early mobilization. Agreement: 87.3%

Do you consider it safe to initiate active or active-assisted early mobilization in a critically ill adult, with or without invasive support, during the administration of a plasma transfusion?
Recommendation: No: it is recommended to wait until the transfusion has finished before initiating early mobilization. Agreement: 91.5%

Monitoring of routine laboratory tests
Regarding routine laboratory tests, how much should hemoglobin decrease over 24 hours to contraindicate the initiation of active or active-assisted early mobilization in critically ill patients, with or without invasive support?
Recommendation: A decrease of ≥ 2 g/dL compared with the previous day's value. Agreement: 91.5%

Regarding routine laboratory tests, how much should the platelet count decrease over 24 hours to contraindicate the initiation of active or active-assisted early mobilization in critically ill patients, with or without invasive support?
Recommendation: A decrease of ≥ 20% compared with the previous day's value. Agreement: 91.5%

SIXTH ROUND - Consensus items: 24

Critically ill patient with predominantly respiratory pathology
• Active early mobilization
Platelets: Between 20,000/µL and 40,000/µL. Agreement: 91.5%
Lactate: ≤ 3mmol/L or 27mg/dL. Agreement: 87.3%
pH: Between 7.35 and 7.45. Agreement: 87.3%
• Active-assisted early mobilization
Lactate: ≤ 3mmol/L or 27mg/dL. Agreement: 87.3%
INR: Between 0.8 and 3.0. Agreement: 87.3%

Critically ill patient with predominantly cardiovascular pathology
• Active early mobilization
Platelets: Between 41,000/µL and 60,000/µL. Agreement: 96.6%
Hemoglobin: ≥ 8g/dL. Agreement: 87.3%
Lactate: ≤ 2mmol/L or 18mg/dL. Agreement: 87.3%
INR: Between 0.8 and 1.5. Agreement: 87.3%
Potassium: Between 3.5mmol/L and 5.0mmol/L. Agreement: 91.5%
• Active-assisted early mobilization
Hemoglobin: ≥ 7g/dL. Agreement: 96.6%
Lactate: ≤ 3mmol/L or 27mg/dL. Agreement: 87.3%
INR: Between 0.8 and 3.0. Agreement: 87.3%
pH: Between 7.35 and 7.45. Agreement: 91.5%

Critically ill patient with predominantly renal pathology
• Active early mobilization
Potassium: Between 3.5mmol/L and 5.0mmol/L. Agreement: 91.5%
Critically ill patient with predominantly surgical pathology
• Active early mobilization
Platelets: Between 41,000/µL and 60,000/µL. Agreement: 87.3%
Lactate: ≤ 2mmol/L or 18mg/dL. Agreement: 87.3%
• Active-assisted early mobilization
INR: Between 0.8 and 1.5. Agreement: 91.5%

Critically ill patient with predominantly hematological/immunological pathology
• Active early mobilization
Lactate: ≤ 2mmol/L or 18mg/dL. Agreement: 100%
• Active-assisted early mobilization
Lactate: ≤ 2mmol/L or 18mg/dL. Agreement: 100%
INR: Between 0.8 and 3.0. Agreement: 87.3%

Critically ill patient with predominantly neurological pathology
• Active early mobilization
Platelets: Between 20,000/µL and 40,000/µL. Agreement: 87.3%
• Contraindication to initiating active and active-assisted early mobilization
Hemoglobin: ≤ 7g/dL. Agreement: 91.5%

Early mobilization in the context of transfusions
Do you consider it safe to initiate active or active-assisted early mobilization in a critically ill adult, with or without invasive support, during the administration of a red blood cell/packed red cell transfusion?
Recommendation: No: it is recommended to wait until the transfusion has finished before initiating early mobilization. Agreement: 91.5%

SEVENTH ROUND - Consensus items: 30

Critically ill patient with predominantly respiratory pathology
• Active-assisted early mobilization
Platelets: Between 20,000/µL and 40,000/µL. Agreement: 100%
pH: Between 7.24 and 7.45. Agreement: 87.3%
• Contraindication to initiating active and active-assisted early mobilization
Lactate: ≥ 3mmol/L or 27mg/dL. Agreement: 91.5%
INR: ≥ 3.1. Agreement: 100%

Critically ill patient with predominantly cardiovascular pathology
• Active-assisted early mobilization
Platelets: Between 41,000/µL and 60,000/µL. Agreement: 87.3%
Potassium: Between 3.5mmol/L and 5.0mmol/L. Agreement: 87.3%
• Contraindication to initiating active and active-assisted early mobilization
Lactate: ≥ 3mmol/L or 27mg/dL. Agreement: 100%

Critically ill patient with predominantly renal pathology
• Active early mobilization
Platelets: Between 41,000/µL and 60,000/µL. Agreement: 87.3%
Lactate: ≤ 3mmol/L or 27mg/dL. Agreement: 100%
• Active-assisted early mobilization
INR: Between 0.8 and 3.0. Agreement: 100%
Lactate: ≤ 3mmol/L or 27mg/dL. Agreement: 87.3%
pH: Between 7.24 and 7.45. Agreement: 87.3%
• Contraindication to initiating active and active-assisted early mobilization
INR: ≥ 3.1. Agreement: 87.3%

Critically ill patient with predominantly surgical pathology
• Active-assisted early mobilization
Platelets: Between 41,000/µL and 60,000/µL. Agreement: 100%
Lactate: ≤ 2mmol/L or 18mg/dL. Agreement: 100%
pH: Between 7.24 and 7.45. Agreement: 100%
• Contraindication to initiating active and active-assisted early mobilization
Lactate: ≥ 2mmol/L or 18mg/dL. Agreement: 96.6%
INR: ≥ 1.6. Agreement: 87.3%

Critically ill patient with predominantly hematological/immunological pathology
• Active early mobilization
Platelets: Between 20,000/µL and 40,000/µL. Agreement: 96.6%
• Active-assisted early mobilization
Platelets: Between 20,000/µL and 40,000/µL. Agreement: 100%
pH: Between 7.35 and 7.45. Agreement: 100%
• Contraindication to initiating active and active-assisted early mobilization
Platelets: ≤ 20,000/µL. Agreement: 96.6%
Lactate: ≥ 2mmol/L or 18mg/dL. Agreement: 96.6%
INR: ≥ 3.1. Agreement: 96.6%

Critically ill patient with predominantly neurological pathology
• Active-assisted early mobilization
Platelets: Between 20,000/μL and 40,000/μL. Agreement: 91.5%
Lactate: ≤ 3mmol/L or 27mg/dL. Agreement: 96.6%
pH: Between 7.24 and 7.45. Agreement: 96.6%
• Contraindication to initiating active and active-assisted early mobilization
Platelets: ≤ 20,000/μL. Agreement: 91.5%
Lactate: ≥ 3mmol/L or 27mg/dL. Agreement: 96.6%
INR: ≥ 1.6. Agreement: 100%

EIGHTH ROUND - Consensus items: 6

Critically ill patient with predominantly respiratory pathology
• Contraindication to initiating active and active-assisted early mobilization
Platelets: ≤ 20,000/μL. Agreement: 96.6%

Critically ill patient with predominantly cardiovascular pathology
• Contraindication to initiating active and active-assisted early mobilization
Platelets: ≤ 40,000/μL. Agreement: 96.6%

Critically ill patient with predominantly renal pathology
• Active-assisted early mobilization
Platelets: Between 20,000/μL and 40,000/μL. Agreement: 96.6%
• Contraindication to initiating active and active-assisted early mobilization
Platelets: ≤ 20,000/μL. Agreement: 96.6%
Lactate: ≥ 3mmol/L or 27mg/dL. Agreement: 96.6%
Critically ill patient with predominantly surgical pathology
• Contraindication to initiating active and active-assisted early mobilization
Platelets: ≤ 40,000/μL. Agreement: 96.6%
